# Distant recurrence and margin involvement in invasive breast cancer

**DOI:** 10.1038/s41416-025-03275-z

**Published:** 2026-01-05

**Authors:** Sarah Michael, John Broggio, Sarah Bowers, Jane Ooi, Anne Armstrong, Mohammed Absar, Nikitas Dimipoulos, Simon Ellenbogen, Jacqui Gath, Glen Martin, Nigel James Bundred

**Affiliations:** 1 University of Manchester, Manchester, UK; 2 Manchester University NHS Foundation Trust, Wythenshawe, Manchester, UK; 3 National Cancer Registration and Analysis Service, NHS England, Manchester, UK; 4 Bolton NHS Foundation Trust, Bolton, Greater Manchester UK; 5 Dept Med Oncology, Christie Hospital, Manchester, UK; 6 Pennine Acute Hospitals NHS Trust, Crumpsall, Manchester, Greater Manchester UK; 7 Tameside and Glossop Integrated Care NHS Foundation Trust, Ashton-under-Lyne, UK; 8 Independent Cancer Patient Voice, London, UK; 9 Division of Informatics, Imaging and Data Science, Faculty of Biology, Medicine and Health, University of Manchester, Manchester Academic Health Science Centre, Manchester, UK

**Keywords:** Surgical oncology, Breast cancer

## Abstract

**Abstract:**

The effect of involved margins after breast cancer surgery on distant recurrence (DR) is unknown. We determined the association between margin width or involvement, DR and cancer deaths.

**Patients and methods:**

Greater Manchester (GM) and the National Cancer Registry (NCRAS) cohorts were analysed. Margin status after curative surgery was measured. Cox-proportional hazards investigated factors associated with LR, DR and breast cancer deaths.

**Results:**

In GM (2010–2014), 2295 (70.2%) patients had clear margins ( > 2 mm), 302 (9.2%) close (1–2 mm) and 673 (20.6%) involved ( < 1 mm) margins. 2030 patients underwent breast conservation surgery (BCS). After multivariable adjustment in BCS patients, involved margins had an increased hazard of DR (HR 1.73, 95% CI:1.03, 2.88, *p* = 0.037) and LR (HR 2.16, 95% CI:1.31, 3.58, *p* = 0.003). NCRAS data from 2010–2013 in 16,420 BCS patients included 3,913 patients (23.9%) with final margins <1 mm. There were 642 deaths (3.9%) after 80.2 months median follow-up: 5.6% in patients with final margins <1 mm and 3.4% with margins >1 mm. After BCS, in 5246 patients who underwent chemotherapy after BCS, involved margins <1 mm had a HR of 1.33 (CI 1.10–1.60, *p* = 0.003) for cancer death.

**Conclusions:**

Margins >1 mm were associated with lower DR and cancer deaths. Guidelines should recommend a minimum margin clearance of 1 mm.

## Introduction

Breast cancer causes 11,400 deaths annually in the UK. Most women with early breast cancer receive conservation surgery (BCS). If cancer cells remain present at the edges (of the cut) there is an increased risk of cancer returning at the same site [1, 2]. Previous studies have shown an association between the width of tumour from the margin in colorectal cancer and subsequent outcomes [1, 2].

Removing cancers without leaving tumour at a surgical margin reduces local recurrence (LR) [3–8] but the effects of margin involvement on distant recurrence (DR) are unclear. How far the tumour should be from the margin is controversial. After BCS, adjuvant treatment including radiotherapy to the breast [3, 7, 9]. and endocrine therapy reduce LR [8, 10, 11].

American Society of Clinical Oncology (ASCO) 2014 guidelines [12], state that negative margins reduced LR but recommended “a margin of no tumour on ink” was sufficient clearance around invasive cancer after BCS [12]. The effect of margin width on DR or breast cancer mortality is unclear [13].

Tumour at the margin (ToI) led to an increased risk of DR (HR 1.75 (1.17–2.62)) in two trials [14]. UK Guidelines require a final cancer margin clearance of 1 mm or more [7, 9] but a prospective study POSH found that the 21% of patients left with margins ( < 1 mm) involved, suffered increased DR and worse survival [8].

Our recent metanalysis of margin status after BCS found a 1 mm margin clearance at the edge of a BCS specimen reduced DR and LR [4]. Randomized trials no longer consider women with involved margins as eligible for study participation.

If DR and cancer deaths are associated with involved surgical margins after treatment, changes to current international practice are required. The aim of this study was to use two large UK breast cancer patient datasets to: 1) determine if margin involvement was associated with DR and death from breast cancer, 2) determine the optimal margin width to reduce DR, LR and breast cancer deaths after BCS.

## Methods

### Greater Manchester (GM) cohort

**Fig. 1:**
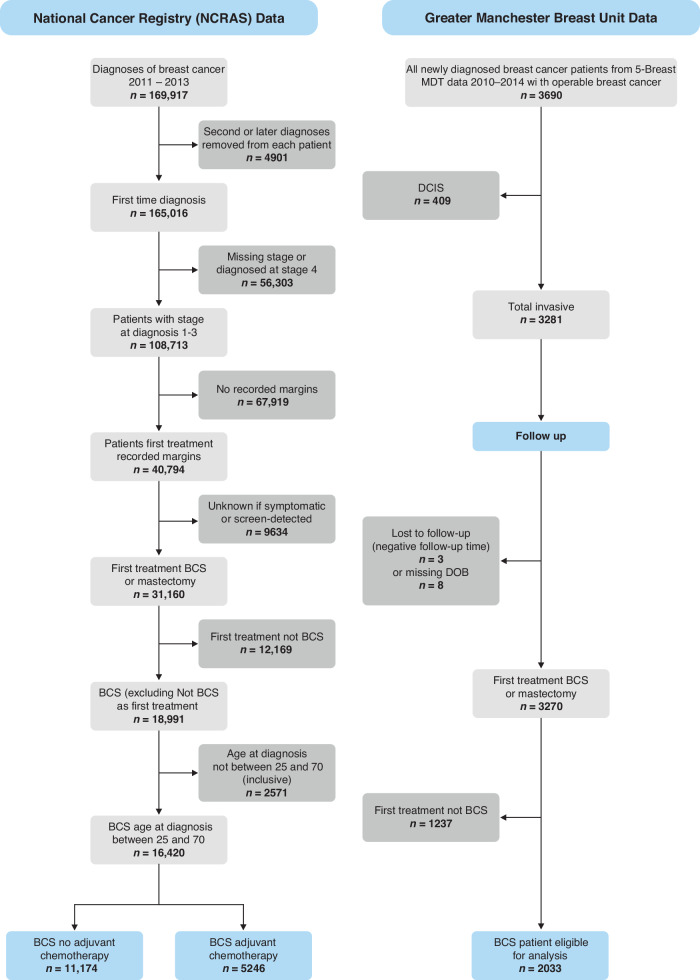
CONSORT Diagram.

An audit in 5 Breast Units of patients diagnosed with early invasive primary breast cancer(T1–3) was undertaken between January 2010 and December 2014. Ethical approval was obtained (IRAS Nos 275022 and 248313) for the research. Patients undergoing neoadjuvant therapy or not undergoing curative surgery, with inoperable, T4, inflammatory or metastatic cancer were excluded (Fig. 1:Supplementary Table 1a). Pathological data (type, size, tumour grade and node status), oestrogen (ER) and progesterone (PR), HER2 receptor status were prospectively recorded on all patients using the National Health Service Breast Screening Pathology (NHSBSP) reporting standards. Final margin status and exact width(mm) were prospectively recorded after surgery (including re-excision) according to NHSBSP standards [15]. Final margins more than 1 mm clearance were considered clear as per local GM and ABS guidelines [9].

Women underwent BCS or mastectomy with either sentinel node biopsy or clearance and then adjuvant radiotherapy, endocrine and chemotherapy according to local guidelines. Patients excluded from radiotherapy included those over 75 years of age, with a cancer <1 mm, low grade or ER positive.

### NCRAS cohort

National Cancer Registry (NCRAS) data analysis received ethics and Public Health England approvals. Data for the years 2010–2013(Fig. 1) included final margin distance (after any re-excision) recorded prospectively from NHSBSP pathology reports as margins >1 mm clear or margins <1 mm. Validation and verification of a subset of NCRAS data were performed. Patients not undergoing surgery (mastectomy or BCS) as primary treatment were not included in the analysis. Other variables available for analysis were age, tumour size, grade, stage, nodal involvement, mode of detection and treatment.

### Outcome definitions (GM & NCRAS cohorts)

The outcomes were LR and DR in the GM data, and breast cancer death in NCRAS data. Within the GM data, LR was defined as histopathological evidence of any recurrence of breast cancer (invasive or DCIS) in the breast, chest wall, or adjacent lymph nodes. DR was defined as a clinically, radiologically, or morphologically verified recurrence (any recurrence in the supraclavicular nodes or beyond) and deaths from breast cancer. Recurrence outcomes were confirmed by review of electronic patient notes and histopathology reports for all patients. Simultaneous LR and DR was defined as pathologically proven LR and DR developing within a 3-month period.

Within NCRAS, cause and timing of death are obtained from death certification (1 A/B) for England. Follow-up was defined from the date-of-surgery in all cases.

### Statistical analysis

Baseline characteristics as a full cohort, and across groups of final margin status, were summarised using the mean, standard deviation and range for continuous variables, and frequencies of occurrence for categorical data.

We fitted a logistic regression model, with margins (clear vs. close/involved) as the outcome, and all the other variables in the dataset as covariates. Associations between the covariates and margin status were summarised through odds ratios (ORs) and 95% confidence intervals.

Within the GM data time-to-LR and time-to-DR analyses were conducted under a competing risk framework (where all-cause death was a competing risk) [16–18]. Univariable analysis of time-to-event outcomes were visually explored using cumulative incidence plots, summarised across margin status. LR and DR rates were cross tabulated by presentation route and by margin status.

To explore whether margin status was associated with recurrence, we fitted cause-specific Cox-proportional hazards models. Associations between patient, tumour, and surgical characteristics on recurrence were estimated both as a whole cohort and in a subset of symptomatic only patients. These associations were quantified using hazard ratios (HR) and associated 95% confidence intervals. The cause-specific Cox proportional hazards models included margin status (close (1.1–2 mm), involved < 1 mm, or clear > 2 mm) and other variables recorded in the dataset. The proportional hazards assumption for the primary variable of interest (margin status) was checked in all Cox models by examining the Schoenfeld residuals. Sensitivity analysis was undertaken for BCS only patients to address margin width according to ASCO and ABS guidelines and for symptomatic patients after analysis demonstrated it as a strong predictive factor for relapse.

Similar analyses were undertaken in the NCRAS data, but outcome changed to death from cancer. Death from any other cause was taken as a competing risk, again employing cause-specific Cox-proportional hazards models to explore the association between margin status and cancer mortality [17, 18]. Statistical analysis was performed using SPSS software, R version 4.0.2 [16, 19] and Stata version 16.1. For R, the following packages were used: tidyverse, MICE [19], survival [20–22] and cmprsk [23].

### Patient and public involvement

Independent Patient Cancer Voice patient representatives felt that our findings were clear, to avoid technical terms and to enable wide dissemination of the results given the implications for research and clinical practice

## Results

### Greater Manchester (GM) cohort

Across GM hospitals, 3281 cases of invasive breast cancer treated (Fig. 1) either by BCS (*n* = 2030) or mastectomy (*n* = 1240) with 11(0.34%) patients excluded due to no follow-up or incorrect patient identifiers leaving 3270 patients, with 1817 (55.6%) presenting symptomatically and 1453 (44.4%) via breast screening.

Median age was 61 (range 24–100) years. Median follow up was 64.4 months (range 0.0–126.6). Most patients underwent adjuvant radiotherapy (71%) and/or hormone therapy (84%). Out of 2033 patients undergoing BCS, 1873 (92.1%) received radiotherapy, but 160 (7.9%) didn’t, mainly due to older age. Radiotherapy was given to 37% patients post-mastectomy. Overall, 3231 (98.8%) patients had adjuvant systemic therapy. Most patients were T-stage 1 (58%) or 2 (37%), with 73% N-stage N0 (Supplementary Table 1) with ER positive (84%), PR positive (73%), and 12% HER2 positive cancers.

### Margin involvement

Overall, 2295 patients (70.2%) had clear ( > 2 mm), 302 (9.2%) close ( > 1–2 mm) and 673 (20.6%) involved final margins ( < 1 mm). Only 193(6.2%) of cancers had “Tumour on ink” (ToI) margins.

Women presenting with symptoms more often had margin involvement than screening presentation (OR 1.39,95% CI: 1.12, 1.72, *p* = 0.002). Amongst BCS patients, mode of presentation and grade predicted margin involvement (Supplementary Tables 4). Margins were potentially re-excisable in 64% of patients (radial or anterior) (Supplementary Table 6a, b).

### Local (LR) and distant (DR) recurrence

**Fig. 2:**
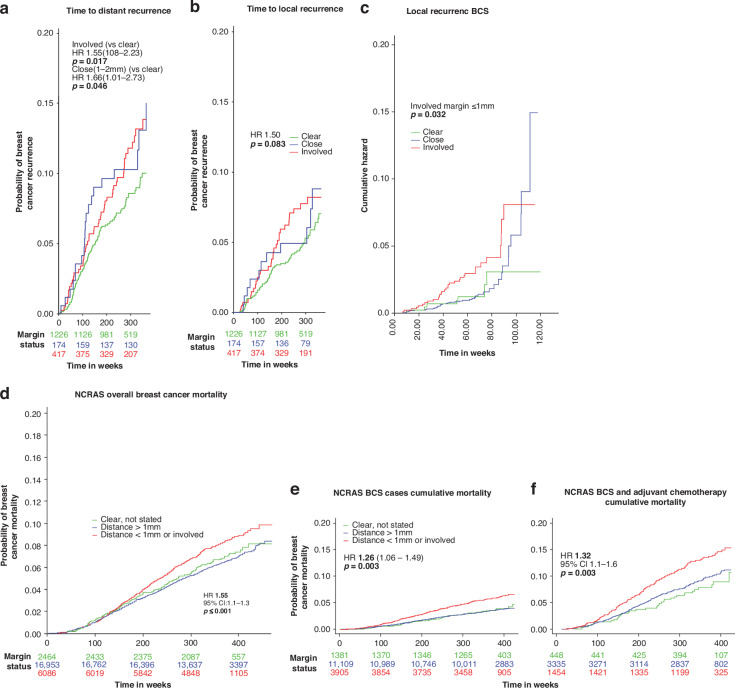
Margin width association with Distant and Local Recurrence. Cumulative incidence functions by prospectively measured margin status for (**a**) distant and (**b**) local recurrence in patients presenting with symptomatic breast cancer in Greater Manchester 2011–2103, each adjusted for the competing risks of death. **c** Involved margin < 1 mm (ToI excluded,0 vs close 1–2 mm versus 2 mm or greater) in GM. **d** National Cancer Registry (NCRAS) 2010–2013 data prospectively assessed surgical widths and Breast Cancer Mortality in 24962 women aged 25–70 years in England. Mortality according to Death Certification (1 A/B) Margin Status. Clear>1 mm; Clear distance not stated compared to involved margins <1 mm. Between 2010 and 2013, in 24962 breast cancers treated surgically on the NCRAS database, 22.6% cancer patients had involved margins <1 mm, 67.8% margins >1 mm and 9.7% were classed as “clear margins” by their MDT as margins>1 mm but exact margin width was not reported. The patients with clear margins were defined by their MDT are shown separately in (**d**, **e**) and it can be seen that Clear margins had a similar survival to Margins>1 mm and significantly different to margins<1 mm. **e** Effect of margins<1 mm (red lines) compared Margins > 1 mm (green) on cancer mortality in 16,420 Breast Conservation Surgery treated NCRAS patients aged 25–70 years in England. **f** NCRAS mortality data of 5624 Breast Conservation Surgery patients who underwent post-op chemotherapy and margin status. Mortality according to Death Certification (1 A/B) Margin Status. Clear>1 mm; Clear distance not stated compared to involved margins <1 mm.

LR developed in 160 (4.9%) and DR in 231 patients (7.1%). Thirty (1.3%) patients experienced simultaneous LR and DR. Table 2 and Fig. 2a show the cumulative incidence functions for LR and DR. Time-to-DR (*p* = 0.017) and time-to-LR (*p* = 0.083) differed by margin status. At 5-years, the probability of DR was 4.84%, 5.9% and 7.14%, for clear>2 mm, close (1–2 mm) and involved margins<1 mm, respectively. For LR, the corresponding 5-year probabilities were 3.1%, 3.53% and 11% respectively. Time to recurrence for involved margins was median 33.8 months for DR and 42.0 months for LR.

In univariable analysis, involved margins had higher DR rates compared with clear margins (HR 1.38, 95% CI: 1.03, 1.86, *p* = 0.032). In a cause-specific Cox proportional hazards model, involved margins < 1 mm had an increased risk of DR compared with clear margins>1 mm (HR 1.39 (95% CI: 1.02, 1.89: *p* = 0.037), taking into account tumour stage, grade and number of involved nodes (Fig. 2a, b; Table 1)

**Table 1:** Cox proportional hazards model for time-to-local-recurrence (left) and time-to-distant-recurrence (right) in all GM breast cancer cases (3182 entered into the analyses excluding missing cases).

Variable	Multivariate LR HR	*p*	Multivariate DR HR	*p*
Margin Clear>2 mm	REF		REF	
Close 1.1–2 mm	1.15 (0.65–2.04)	0.630	1.15 (0.72–1.84)	0.555
Involved Margin ≤1 mm	1.69 (1.18–2.44)	**0.005**	1.39 (1.02–1.89)	**0.037**
Age at Diagnosis	0.99 (0.98–1.00)	0.121	1.01 (0.99–1.02)	0.361
Radiotherapy	0.95 (0.64–1.41)	0.802	0.83 (0.60–1.15)	0.254
Chemotherapy	0.85 (0.53–1.35)	0.489	0.98 (0.67–1.43)	0.896
Hormone therapy	0.70 (0.33–1.47)	0.339	0.48 (0.26–0.88)	**0.019**
Herceptin therapy	0.78 (0.22–2.76)	0.696	0.53 (0.22–1.30)	0.166
Symptomatic vs. screening	1.96 (1.24–3.10)	**0.004**	1.77 (1.20–2.61)	**0.004**
ER Positive	0.57 (0.08–4.25)	0.584	1.22 (0.17–8.98)	0.848
PR Positive	0.67 (0.41–1.10)	0.110	0.71 (0.49–1.05)	0.086
HER2 Positive	2.35 (0.37–14.81)	0.363	1.39 (0.34–5.70)	0.646
Molecular Subtype				
Triple Negative	REF		REF	
HER2 enriched ER Negative	0.55 (0.13–2.29)	0.407	1.51 (0.48–4.77)	0.485
ER Positive HER2 Negative	0.15 (0.01–2.08)	0.158	0.60 (0.06–6.48)	0.675
ER Positive HER2 Positive	0.80 (0.10–6.45)	0.831	0.95 (0.13–7.28)	0.962
T-Stage 1 (*0–2 cm)*	REF		REF	
T-Stage 2 *(2.1cm-5cm)*	1.45 (0.95–2.22)	0.085	1.68 (1.17–2.40)	**0.005**
T-Stage 3 *(* > *5 cm)*	1.34 (0.54–3.29)	0.527	2.04 (1.11–3.73)	**0.021**
Number of positive nodes				
Negative (0)	REF		REF	
1–3	1.42 (0.95–2.13)	0.089	1.89 (1.34–2.68)	**<0.001**
4–9	1.22 (0.62–2.43)	0.565	3.44 (2.22–5.34)	**<0.001**
10+	2.41 (1.24–4.67)	**0.009**	4.60 (2.88–7.35)	**<0.001**
Size (mm)	1.01 (0.99–1.02)	0.432	1.01 (1.00–1.02)	**0.010**
Tumour Grade 1	REF		REF	
Tumour Grade 2	1.21 (0.63–2.32)	0.575	3.78 (1.50–9.55)	**0.005**
Tumour Grade 3	1.36 (0.67–2.78)	0.393	5.429 (2.11–13.971)	**<0.001**
Lymphovascular invasion	1.71 (1.18–2.49)	**0.005**	1.28 (0.95–1.73)	0.111

Adjusted hazard ratios (HR) and associated 95% confidence intervals. Median time to recurrence for involved margins was 33.8 months for DR and 42.0 months for LR.

Bold values represent the analyses that produced a clinically and statistically significant finding.

All variables shown above were entered into the multivariate.

NB: site of treatment did not affect time to recurrence and is not shown as it was not significant.

Additional data excluding TOI was performed and is shown in Supplementary Tables 7, 8.

Involved (compared to clear>1 mm) margins were associated with higher LR (HR 1.61, 95% CI: 1.14, 2.27, *p* = 0.007). Cox proportional hazards models for LR showed involved margins<1 mm (compared to clear) were associated with higher LR with an adjusted HR 1.69 (95% CI: 1.18, 2.44, *p* = 0.005).

Rates of LR (HR 1.96, 95% CI: 1.24, 3.10, *p* = 0.004). and DR (HR 1.77, 95% CI: 1.12, 2.61, *p* = 0.004) were higher in patients presenting with symptomatic cancer compared with a screening diagnosis (*p* = **<**0.001 (Table 1: Supplementary Table 2).

### Sensitivity analysis: GM breast conservation surgery cases (Table 2)

In Cox Regression analysis of BCS patients, margins <1 mm were associated with increased DR, HR 1.73 (1.03–2.89) and LR, HR 2.16 (1.31–3.58) as were node positivity, grade 3 tumours, HR 8.71 (1.91–39.62), use of endocrine therapy HR 0.25 (0.01–0.63) and radiotherapy HR 0.50 (0.25–0.99) (Table 2).

**Table 2:** Time-to-recurrence in Breast Conserving patients in GM. Adjusted hazard ratios (HR) and associated 95% confidence intervals (1982 patients entered in the analysis to exclude missing cases.

Variable	Multivariate LR HR	*p*	Multivariate DR HR	*p*
Margin Clear>2 mm	REF		REF	
Close 1.1–2 mm	0.98 (0.42–2.27)	0.963	1.45 (0.72–2.95)	0.302
Involved Margin ≤1 mm	2.16 (1.31–3.58)	**0.003**	1.73 (1.03–2.88)	**0.037**
Age at Diagnosis	0.99 (0.97–1.01)	0.156	0.99 (0.97–1.01)	0.416
Radiotherapy	0.50 (0.24–1.00)	0.061	0.50 (0.25–0.99)	**0.048**
Chemotherapy	0.70 (0.34–1.46)	0.338	0.59 (0.30–1.17)	0.132
Hormone therapy	0.35 (0.13–0.96)	**0.040**	0.25 (0.10–0.63)	**0.003**
Herceptin therapy	3.69 (0.59–23.14)	0.163	0.67 (0.11–3.87)	0.649
Symptomatic vs. screening	1.94 (1.00–3.74)	**0.049**	1.59 (0.79–3.17)	0.191
ER Positive	3.58 (1.19–10.78)	**0.023**	2.56 (0.91–7.23)	0.076
PR Positive	0.39 (0.18–0.62)	**<0.001**	0.69 (0.37–1.28)	0.235
HER2 Positive	0.21 (0.03–1.42)	0.109	1.15 (0.21–6.26)	0.871
T-Stage 1 (*0–2 cm)*	REF		REF	
T-Stage 2 *(2.1cm-5cm)*	2.32 (1.08–4.97)	**0.031**	1.69 (0.82–3.52)	0.158
T-Stage 3 *(* > *5 cm)*	2.81 (0.32–24.31)	0.349	0.76 (0.09–6.37)	0.804
No. of nodes				
Negative (0)	REF		REF	
1–3	1.34 (0.73–2.48)	0.349	2.62 (1.47–4.66)	**0.001**
4–9	0.93 (0.27–3.19)	0.912	5.26 (2.45–11.29)	**<0.001**
10+	0.78 (0.17–3.63)	0.753	2.77 (0.95–8.07)	0.062
Size (mm)	0.98 (0.94–1.03)	0.420	1.027 (0.99–1.07)	0.169
Tumour Grade 1	REF		REF	
Tumour Grade 2	1.42 (0.63–3.21)	0.398	4.28 (0.99–18.59)	0.052
Tumour Grade 3	1.71 (0.66–4.34)	0.266	8.71 (1.91–39.62)	**0.005**
Lymphovascular invasion	1.66 (0.94–2.94)	0.080	1.09 (0.62–1.90)	0.776

Median time to DR for involved margins was 33.8 months and 42.0 months for LR.

Bold values represent the analyses that produced a clinically and statistically significant finding.

All variables shown above were entered into the multivariate.

NB: site of treatment has been hidden as it did not affect time to recurrence and was not significant.

Additional data excluding TOI was performed and is shown in Supplementary Tables 7, 8.

Involved margins were independently associated with LR even when ToI cases were excluded from the analysis HR 1.56 (1.10–2.38, *p* = 0.038) (Fig. 2c: Supplementary Tables 7, 8). Restricting analysis to BCS patients who underwent chemotherapy found margins <1 mm had a HR of 1.71 (1.17–2.50: *p* = 0.005) for DR and for margins 1–2 mm a HR of 1.78 (1.03–3.09: *p* = 0.041) compared to margins<2 mm. Increased LR was associated with margins <1 mm, HR 1.84 (1.15–2.94: *p* = 0.041). The majority of breast cancer deaths in GM patients occurred in Stage 1 or 2 cancers (Supplementary Table 5).

### NCRAS cohort

Between 2010 and 2013, out of 40,794 breast cancers treated surgically on the NCRAS database aged 20–112 years old, 9,210 (22.6%) cancer patients had involved margins <1 mm, 27,644 margins >1 mm (67.8%) and 3,940 (9.7%) were classed as “clear margins” by their MDT but exact margin width was not reported. Median follow-up of patients was 79.9 months (range 0.0–109.2) with 3,176 (7.8%) breast cancer deaths. Margins were involved in 23.9% patients whose first treatment was BCS and 16.9% of patients when it was mastectomy (Supplementary Table 9).

An unadjusted excess cancer mortality occurred in the 22.6% of patients whose pathology indicated margins<1 mm (HR 1.17 (95% CI 1.08–1.27: *p* < 0.001)) compared to either clear margins>1 mm or margins coded as “clear” without a specific margin width recorded. Absolute cancer death rates were 7.5% with clear to 8.7% with involved margins 5 years after surgery. In multivariate analysis margins remained associated with cancer mortality (HR 1.15 (1.06–1.25: *p* = 0.001)). Combining margins >1 mm and “clear” margins groups compared to patients with involved margins, the HR was 1.17 (1.08–1.27 (*p* < 0.001)). Restricting analysis to patients aged 18–65 years or 18–50 years reduced patient numbers but 1617 cancer deaths occurred and involved margins remained in multivariate analysis (HR 1.21 (1.08–1.35), *p* < 0.001: Fig. 2d).

### NCRAS BCS patients

Overall, 18,991 patients aged 25–70 years in England underwent BCS, 4534 of whom had final margins <1 mm (23.9%). In multivariate analysis, patients with final margins <1 mm were associated with a worse survival (HR 1.22 (1.05–1.42: *p* = 0.009)).

Analysing the 16,420 patients aged 25–70 years old whose first cancer treatment was BCS, there were 3,696 patients with final margins <1 mm and 642 deaths (3.9%) by the end of follow-up: 5.6% in patients with final margins <1 mm and 3.4% with margins >1 mm, respectively. Margins<1 mm remained significant in multivariate Cox regression analysis (Fig. 2e: Supplementary Table 9). Absolute cancer death rates increased from 7.5% with clear margins to 8.7% with involved margins 5 years after surgery.

Multivariate analysis showed margins<1 mm (HR 1.28 (1.08–1.52): *p* = 0.004) were associated with increased cancer deaths along with tumour stage (stage 3 HR 5.18 (4.05–6.64, *p* < 0.001)), Grade 3, HR 8.91 (5.86–13.56, *p* < 0.001), age (HR 1.03 (1.02–1.04, *p* < 0.001), symptomatic detection (HR 1.91 (1.61–2.27, *p* < 0.001) and lower socio-economic status HR 1.33 (1.02–1.73, *p* < 0.001) (Table 3).

**Table 3:** National Cancer registry: Multivariable analysis of Factors affecting Breast Cancer Death in 16,420 women aged 25–70 years who received BCS as a part of their treatment.

Factor	Frequency	*N*	Margins < 1 mm	Multivariate	95%CI		*P* Value
Margins	Margins >1 mm	11126	0.0%	REF			
	“Clear”	1381	0.0%	0.93	0.69	1.25	*p* = 0.637
	Margin<1 mm	3913	100.0%	1.28	1.08	1.52	*p* = 0.004
Detection method	Symptomatic	5900	28.4%	1.91	1.61	2.27	*p* < 0.001
	Screen detected	10520	21.3%	REF			
Age	Age increase/year	16420	23.8%	1.03	1.02	1.04	*p* < 0.001
Deprivation Status	Least (1)	4044	25.6%	1.06	0.84	1.34	*p* = 0.595
	2	4154	24.4%	1.03	0.81	1.29	*p* = 0.834
	3	3635	22.9%	REF			
	4	2577	22.5%	1.22	0.95	1.57	*p* = 0.124
	Most deprived (5)	2010	22.7%		1.02	1.73	*p* = 0.035
Stage of Ca	Stage 1	11059	21.1%	REF			
	Stage 2	4845	28.8%	1.99	1.67	2.38	*p* < 0.001
	Stage 3	516	36.2%	5.18	4.05	6.64	*p* < 0.001
Grade	1	3746	18.4%	REF			
	2	8073	23.6%	3.01	1.97	4.61	*p* < 0.001
	3	4429	29.0%	8.91	5.86	13.56	*p* < 0.001
	Unknown	172	19.2%	2.38	0.72	7.90	*p* = 0.158

*All variables shown above were entered into the multivariate*. The accepted approach to using death certification for analysis was applied, 1 A/B death certification meaning that breast cancer directly caused the patients death whereas deaths classified 2/3 were because the patient with breast cancer but had another cause of death.

Restricting analysis to 5246 patients aged 25–70 years whose first treatment was BCS followed by adjuvant chemotherapy, there were 1457 patients with final margins <1 mm.

After follow-up 520 deaths (9.9%) had occurred: 12.5% in patients with final margins <1 mm and 9.1% in patients with margins >1 mm. Margins <1 mm were associated with increased breast cancer mortality in multivariate analysis (HR 1.33 (1.10–1.60): *p* = 0.003) (Fig. 2f) (Table 4).

**Table 4:** National Cancer Registry: Multivariable analysis of Factors affecting Breast Cancer Death in 5246 women aged 25–70 years who received BCS and adjuvant chemotherapy as their treatment.

Factor	Frequency	*N*	Margins<1 mm	Multivariate	95%CI		*P* Value
Margins	Margins >1 mm	3341	0.0%	REF			
	“Clear”	448	0.0%	0.84	0.59	1.19	*p* = 0.314
	Margin<1 mm	1457	100.0%	1.33	1.10	1.60	*p* = 0.003
Detection method	Symptomatic	2791	30.2%	1.71	1.42	2.07	*p* < 0.001
	Screen detected	2455	25.0%	REF			
Age	Age increase/year	5246	27.8%	1.04	1.03	1.06	*p* < 0.001
Deprivation Status	Least (1)	1247	29.6%	1.09	0.84	1.40	*p* = 0.528
	2	1298	29.2%	0.98	0.76	1.27	*p* = 0.893
	3	1175	27.3%	REF			
	4	830	24.8%	1.15	0.87	1.53	*p* = 0.322
	Most deprived (5)	696	26.2%	1.20	0.90	1.61	*p* = 0.211
Stage of Ca	Stage 1	2037	24.5%	REF			
	Stage 2	2746	28.6%	1.12	0.92	1.36	*p* = 0.266
	Stage 3	463	37.6%	2.09	1.60	2.73	p < 0.001
Grade	1	288	21.9%	REF			
	2	1973	26.2%	1.59	0.90	2.81	p = 0.108
	3	2954	29.5%	2.40	1.38	4.18	p = 0.002
	Unknown	31	19.4%	1.78	0.51	6.26	p = 0.369

*All variables shown above were entered into the multivariate*. The accepted approach to using death certification for analysis was applied, 1 A/B death certification meaning that breast cancer directly caused the patients death whereas deaths classified 2/3 were because the patient with breast cancer had another cause of death.

In 5,900 symptomatic patients aged 25–70 years treated by BCS first: 1673 patients had final margins <1 mm and 408 deaths (6.9%) occurred on follow-up: 9.1% in patients with final margins <1 mm and 6.1% in patients with margins >1 mm. Margins <1 mm remained in the multivariate Cox analysis (HR 1.33 (1.08–1.63): *p* = 0.007) (Supplementary Table 10).

## Discussion

BCS patients with a margin of less than 1 mm after their final surgery had higher subsequent DR and cancer death in comparison to patients with clear margins >1 mm. This association was independent of tumour stage, grade, receptor status and use of adjuvant chemotherapy. Involved margins were associated with LR after GM patients with ToI margins were excluded from the analysis. These findings support evidence from a published metanalysis in which margins<1 mm were associated with 4.9% more LR and higher DR [4].

## Strengths and limitations

This is the largest cohort study addressing surgical margins with final margin data assessed prospectively using standard pathology reporting criteria. Several limitations should be noted when interpreting the results from this study. Firstly, this was not randomised trial data and therefore the presence of unobserved confounding remains and the results cannot be confirmed as causal. Margin width data was missing from some NCRAS registered patients and NCRAS does not have LR data. Similar levels of margin involvement (1 mm) were found in a prospective UK study [8] and we verified Coding from NHSBSP pathology reports in a large sample of NCRAS patients. The nature of the margin involvement (invasive cancer or DCIS) could not be ascertained, but previous studies found both DCIS or invasive cancer at any margin and any location of involvement increased the development of LR [24, 25]. An association with cancer-related mortality beyond ten years could not be studied. Although the absolute effect of involved margins appears small, it was associated with 3% more DR and 3.4% higher cancer mortality. The development of DR, even with modern therapeutic approaches, results in incurable disease. The adverse impact of margin involvement on LR and DR after BCS persisted despite the use of adjuvant chemotherapy.

## Interpretation of findings with existing literature

One in four women who develop LR within 5 years of surgery die within 15 years from breast cancer [6]. Although re-excision of involved margins is advised [7, 9, 12], the definition of involved margins varies internationally [7, 9, 12]. LR ranged between 3.8–8% at 5 years in symptomatic cancers treated in GM. NCRAS does not have LR data, although rates of LR have fallen in clinical trials; randomised trials exclude recruitment of patients with involved margins [11]. DR was more frequent and occurred without LR after 64 months followup (Supplementary Table 5), implying margin involvement can contribute to metastatic disease without causing LR first. Persistence of incompletely removed breast cancer in the breast is a potential source of post-surgery metastatic dissemination [26, 27]. Residual margin involvement <1 mm should lead to re-excision because up to 60% of patients with DCIS and invasive cancer and 36% with invasive disease have further cancer in the re-excision specimen [27]. Boost dose radiotherapy after BCS reduced LR but did not reduce distant recurrence from breast cancer after 10 years of follow-up [28]. Surgical margins were associated with increased risks of DR, independently of whether LR developed and LR occurred less frequently than DR. In BCS patients with margins<1 mm given adjuvant chemotherapy, cancer mortality remained higher than those with clear margins>1 mm, demonstrating adjuvant chemotherapy does not fully compensate for incomplete excision of a cancer.

The margin width necessary to ensure complete surgical removal remains controversial [3, 4, 7, 8, 29]. ASCO guidelines state a “margin of no tumour on the edge of the inked” specimen is sufficient clearance after BCS to reduce LR [12] based on a metanalysis of 28,000 patients undergoing BCS and radiotherapy in 33 studies from 1979–1985. Most patients in the ASCO metanalysis did not receive systemic adjuvant therapy [12], and the study did not use a specific definition of close or negative margins, instead considering any margin greater than ToI was negative. However, our recent metanalysis [4] and this data suggest that at least a 1 mm margin is required to reduce DR, LR and cancer mortality even with current adjuvant therapies. Indeed, involved margins (excluding ToI) were associated with increased LR in this study. The current best evidence for ASCO guidelines needs updating.

An EBCTCG metanalysis [11] found reductions in DR in early breast cancer between 1990 and 2009, was explained by a greater proportion of women with lower-risk disease(node negative) entering trials and adjuvant treatment. The absolute benefit of adjuvant therapy on DR was 2.8% in ER positive cancers. In the USA and UK, the majority of breast cancer deaths now occur in Stage 1/2 ER positive, node negative patients [30] (as in the GM data), a group already prescribed adjuvant endocrine therapy, indicating the potential extra therapeutic benefit for complete surgical excision leading to reductions in DR and cancer deaths.

Both BCS cohorts used measured margin data from a standardised NHSBSP minimum dataset in patients treated with systemic adjuvant therapy to demonstrate that a final margin <1 mm is associated with an increased risk of DR and cancer mortality. The margin width used to ensure tumour clearance should minimise DR. Decisions about re-excision to clear margins should be a consensual decision between clinicians and patients [31]. A previous IPCV member survey [4] indicated patients prefer full information about margins and that decisions about margin widths and re-excision must be a consensual decision between clinicians and patients with full disclosure of the risks of increased DR associated with close margins [3, 4, 26, 28].

BCS patients with symptomatic cancers had a worse prognosis than screen detected cancers as expected but involved margins independently produced worse outcomes.

Countries which do not audit margin status have higher rates of margin involvement and LR [10, 11] which may reflect poor surgical quality.

International guidelines should aim to minimise DR and cancer deaths by recommending complete cancer excision( > 1 mm) after BCS [4, 5, 8, 24].

## Conclusion

In early breast cancer, where surgery is the main driver of cure, accepting the possibility of unobserved confounding in our analysis, incomplete excision may contribute to breast cancer mortality in good prognosis cancers.

## Research in context

*Evidence before this study*

Guidelines for women undergoing breast conserving surgery (BCS) for early invasive cancer recommend avoiding tumour on ink to reduce local recurrence (LR) risk, the effect of margin clearance on cancer deaths is unknown.

*Added value of this study*

In cohort studies using prospectively assessed margin data, BCS patients with a final margin of <1 mm post-surgery had an increased risk of Distant Recurrence (HR 1.73), LR (HR 2.16) and cancer death (HR1.28), independent of tumour stage, grade, receptor status and adjuvant therapy.

*Implications for practice*

International guidelines should minimise DR and cancer deaths by complete cancer excision ( > 1 mm) after BCS.

## Supplementary information

Lo margins supplemental material PDF

## Data Availability

The data and materials are all available through writing to NJB or GM.
