# Supplementary material for: Distant recurrence and margin involvement in invasive breast cancer
Source: Br J Cancer. 2026 Jan 5;134(5):772–80. doi: 10.1038/s41416-025-03275-z (PMC12905278; doi:10.1038/s41416-025-03275-z)
Supplement: Supplementary file 1 — Lo margins supplemental material PDF [file 41416_2025_3275_MOESM1_ESM.pdf]

Supplementary Material

Data on Greater Manchester patients and demographics of cohort.

Greater Manchester Population (GM cohort)

The Greater Manchester (GM) Pathway Board undertook an audit for early breast cancer (T1-3) in five Breast Units. Ethical approval was obtained for subsequent research on the data. All patients in GM diagnosed with invasive primary breast cancer, between January 2010 and December 2014 were included. Patients undergoing neoadjuvant therapy or not undergoing curative surgery, with inoperable, T4, inflammatory or metastatic cancer were excluded (**Figure 1**). Pathological data (type, size, grade and node status), oestrogen (ER) and progesterone (PR), HER2 receptor status and tumour grade were prospectively recorded on all patients using the National Health Service Breast Screening Pathology (NHSBSP) reporting standards. Final margin status and width was prospectively recorded after surgery (including re-excision) according to NHSBSP minimum pathology data standards (15) and final margins more than 1mm clearance considered as clear as per local GM and ABS guidelines (10). GM data provided margin width as Tumour on Ink, <1mm or the exact margin greater than 1mm.

All patients underwent adjuvant radiotherapy in the Christie Cancer Centre where radiotherapy boost to tumour bed was standard of care. Of the 3270 patients,1817 (55.6%) presented symptomatically and 1453 (44.4%) via breast screening.

Supplementary Table 1a: Baseline summary as a full cohort, and across groups of margin status. (GM cohort)

		**Margin Status**			
**Variable**	**Overall**, N = 3,270	**Clear (>2mm)**, N = 2,295[1]	**Close (1.1-2mm)**, N = 302[1]	**Involved (≤1mm)**, N = 673[1]	**p-value**[2]
**Age at Diagnosis**	61 (13) [24, 100]	61 (13) [24, 100]	60 (13) [31, 89]	60 (14) [24, 99]	0.2
**Radiotherapy**	2,334 (71%)	1,536 (67%)	249 (82%)	549 (82%)	**<0.001**
**Chemotherapy**	1,265 (39%)	885 (39%)	100 (33%)	280 (42%)	**0.041**
**Hormone therapy**	2,762 (84%)	1,933 (84%)	263 (87%)	566 (84%)	0.4
**Herceptin therapy**	382 (12%)	270 (12%)	28(9.3%)	84(12%)	0.3

Variable	Overall, N = 3,270	Margin Status			p-value[2]
		**Clear (>2mm)**, N = 2,295[1]	**Close (1.1-2mm)**, N = 302[1]	**Involved (≤1mm),** N = 673[1]	
**Presentation**					**<0.001**
Screening	1,453 (44%)	1,069 (46.6%)	128 (42.4%)	256 (37.9%)	
Symptomatic	1,817 (56%)	1,226 (53.4%)	174 (57.6%)	417 (62.1%)	
**ER Positive**	2,749 (84%)	1,928 (84%)	260 (86%)	561 (83%)	0.6
**PR Positive**	2,375 (73%)	1,665 (73%)	227 (76%)	483 (72%)	0.5
**HER2 Positive**	392 / 3,243 (12%)	274 / 2,271 (12%)	31 / 302 (10%)	87 / 670 (13%)	0.5
**Phenotype**					0.5
Triple Negative	365 / 3,255 (11%)	252 / 2,282 (11%)	31 / 302 (10%)	82 / 671 (12%)	
HER2 enriched ER Negative	138 / 3,255 (4.2%)	102 / 2,282 (4.5%)	10 / 302 (3.3%)	26 / 671 (3.9%)	
ER Positive HER2 Negative	2,468 / 3,255 (76%)	1,736 / 2,282 (76%)	238 / 302 (79%)	494 / 671 (74%)	
ER Positive HER2 Positive	284 / 3,255 (8.7%)	192 / 2,282 (8.4%)	23 / 302 (7.6%)	69 / 671 (10%)	
**T Stage 1** *(0-2cm)*	1,905 / 3,270 (58%)	1,355 / 2,295 (59%)	188 / 302 (62%)	362 / 673 (54%)	**0.029**
**T Stage 2** *(2.1cm-5cm)*	1,210/ 3,270 (37%)	841 / 2,295 (37%)	101 / 302 (33%)	268 / 673 (40%)	
**T Stage 3** *(>5cm)*	155 / 3,270 (4.7%)	99 / 2,295 (4.3%)	13 / 302 (4.3%)	43 / 673 (6.4%)	

Variable	Overall, N = 3,270	Margin Status: Clear (>2mm), N = 2,295[1]	Close (1.1-2mm), N = 302[1]	Involved (≤1mm), N = 673[1]	p-value[2]
**Node Status**					0.6
Negative	2,373 (73%)	1,654 (72%)	220 (73%)	499 (74%)	
1-3 Positive	624 (19%)	443 (19%)	62 (21%)	119 (18%)	
4-9 Positive	169 (5.2%)	127 (5.5%)	10 (3.3%)	32 (4.8%)	
10+Positive	104 (3.2%)	71 / (3.1%)	10 (3.3%)	23 (3.4%)	
**Tumour Size (mm) Mean(Median)[range]**	21 (15) [0, 180]	21 (15) [0, 140]	20 (14) [2,120]	24 (18) [0, 180]	**<0.001**
**Tumour Grade**					0.2
Grade 1	605 / 3,257 (19%)	445 / 2,285 (19%)	48 / 302 (16%)	112 / 670 (17%)	
Grade 2	1,492 / 3,257 (46%)	1,044 / 2,285 (46%)	146 / 302 (48%)	302 / 670 (45%)	
Grade 3	1,160 / 3,257 (36%)	796 / 2,285 (35%)	108 / 302 (36%)	256 / 670 (38%)	
[1] Statistics presented: mean (SD) [minimum, maximum]; n / N (%)	[2] Statistical tests performed: Kruskal-Wallis test; chi-square test of independence; Fisher's exact test				

Patient baseline characteristics as a full cohort, and across groups of margin status (clear, close, and involved) were summarised using the mean, standard deviation and range for continuous variables, and using frequencies of occurrence for categorical data. NB: The only data missing from the analysis was for ER/PR/Her2 status in 10,16 and 54 patients respectively. Total numbers in each group provided where there are missing values in cells.

Supp Table 1b: Baseline summary for BCS patients, and across groups of margin status. (GM cohort)

Variable	Overall, N = 2030	Margin Status			p-value[2]
		Clear (>2mm), N = 1330[1]	Close (1.1-2mm), N = 226[1]	Involved (≤1mm), N = 474[1]	
**Age at Diagnosis Mean(median)[range]**	60.2 (60.8) [23.9, 99.2]	59.9 (60.8) [24.6, 92.5]	60.8 (61.5) [31.3, 89]	60.7 (62) [23.8, 99.2]	0.2
**Radiotherapy**	1873 (92.3%)	1,229 (92.4%)	212 (93.8%)	432 (91.1%)	0.443
**Chemotherapy**	632 (31.1%)	404 (30.4%)	60 (26.5%)	168 (35.4%)	**0.035**
**Hormone therapy**	1740 (85.7%)	1143 (85.9%)	200 (88.5%)	397 (83.8%)	0.227
**Herceptin therapy**	176 (8.7%)	110 (8.3%)	18 (8%)	48 (10.1%)	0.432
**Presentation**					**<0.001**
Screening	1113 (54.8%)	791 (59.5%)	114 (50.4%)	208 (43.9%)	
Symptomatic	917 (45.2%)	539 (40.5%)	112 (49.6%)	266 (56.1%)	
**ER Positive**	1745 (86%)	1151 (86.6%)	198 (87.6%)	396 (83.7%)	0.23
**PR Positive**	1536 (75.8%)	1019 (76.7%)	172 (76.4%)	345 (72.9%)	0.248
**HER2 Positive**	188 / 2014 (9.3%)	119 / 1316 (9%)	20 / 226 (8.8%)	49 / 472 (10.4%)	0.668
**Phenotype**					0.537
Triple Negative	219 / 2018 (10.9%)	137 / 1320 (10.4%)	21 / 226 (9.3%)	61 / 472 (12.9%)	
HER2 enriched ER Negative	56 / 2018 (2.8%)	36 / 1320 (2.7%)	6 / 226 (2.7%)	14 / 472 (3.0%)	

**Variable**	**Overall**, N = 2030	**Margin Status**			p-value[2]
		**Clear (>2mm)**, N = 1330[1]	**Close (1.1-2mm)**, N = 226[1]	**Involved (≤1mm),** N = 474[1]	
ER Positive HER2 Negative	1589 / 2018 (78.7%)	1051 / 1320 (79.6%)	183 / 226 (81%)	355 / 472 (75.2%)	
ER Positive HER2 Positive	154 / 2018 (7.6%)	96 / 1320 (7.3%)	16 / 226 (7.1%)	42 / 472 (8.9%)	
**T Stage 1** *(0-2cm)*	1472 (72.5%)	1006 (75.6%)	164 (72.6%)	302 (63.7%)	**<0.001**
**T Stage 2** *(2.1cm-5cm)*	539 (26.6%)	311 (23.4%)	62 (27.4%)	166 (35%)	
**T Stage 3** *(>5cm)*	19 (0.9%)	13 (1%)	0 (0%)	6 (1.3%)	
**Node Negative**	1662 (81.9%)	943 (72%)	220 (73%)	499 (74%)	0.663
Nodes 1-3 Positive	288 (14.2%)	443 (19%)	62 (21%)	119 (18%)	
4-9 Positive	14 (3%)	127 (5.5%)	10 (3.3%)	32 (4.8%)	
10+Positive	104 (3.2%)	71 / (3.1%)	10 (3.3%)	23 (3.4%)	
**Tumour Size (mm)** **Mean(Median)[range]**	16.8 (15) [0, 140]	16.1 (15) [0, 140]	16.5 (15) [3, 42]	18.6 (17) [0, 75]	**<0.001**
**Tumour Grade** 1	489 / 2024 (24.2%)	357 / 1326 (26.9%)	42 / 226 (18.6%)	90 / 472 (19.1%)	**0.002**
Grade 2	912 / 2024 (45.1%)	579 / 1326 (43.7%)	111 / 226 (49.1%)	222 / 472 (47%)	
Grade 3	623 / 2024 (30.8%)	390 / 1326 (29.4%)	73 / 226 (32.3%)	160 / 472 (33.9%)	
[1] Statistics presented: mean (SD) [minimum, maximum]; n / N (%)	[2] Statistical tests performed: Kruskal-Wallis test; chi-square test of independence; Fisher's exact test				

Supplementary Table 2 Adjusted and unadjusted hazard ratios (HR) and 95% confidence intervals for the cause-specific Cox proportional hazards model for time-to-local-recurrence (left) and time-to-distant-recurrence (right) in GM symptomatic cases only.

Variable	Multivariate LR HR	p	Multivariate DR HR	p
Margin Clear>2mm	REF		REF	
Close 1.1-2mm	1.10 (0.59-2.06)	0.769	1.42 (0.87-2.30)	0.157
Involved Margin ≤1mm	1.45 (0.95-2.20)	0.085	1.55 (1.10-2.18)	**0.011**
Age at Diagnosis	0.99 (0.98-1.01)	0.264	1.00 (0.99-1.02)	0.642
Radiotherapy	1.21 (0.77-1.90)	0.421	0.88 (0.61-1.27)	0.500
Chemotherapy	0.98 (0.58-1.66)	0.937	0.98 (0.64-1.50)	0.927
Hormone therapy	0.51 (0.22-1.15)	0.105	0.76 (0.39-1.50)	0.431
Herceptin therapy	0.70 (0.16-3.15)	0.643	0.64 (0.22-1.87)	0.411
ER Positive	1.11 (0.45-2.74)	0.816	0.99 (0.48-2.02)	0.969
PR Positive	0.67 (0.39-1.18)	0.164	0.69 (0.45-1.05)	0.089
HER2 Positive	1.16 (0.26-5.12)	0.846	1.21 (0.42-3.47)	0.720
T-Stage 1 *(0-2cm)*	REF		REF	
T-Stage 2 *(2.1cm-5cm)*	1.49 (0.93-2.40)	0.101	1.58 (1.05-2.36)	**0.027**
T-Stage 3 *(>5cm)*	1.36 (0.53-3.51)	0.528	1.78 (0.93-3.39)	0.080
Number of positive nodes				
0	REF		REF	
1-3	1.52 (0.96-2.39)	0.072	1.83 (1.24-2.70)	**0.002**
4-9	1.34 (0.66-2.73)	0.412	3.09 (1.88-5.07)	**<0.001**
10+	2.65 (1.33-5.27)	**0.005**	5.01 (3.06-8.20)	**<0.001**
Size (mm)	1.00 (0.99-1.02)	0.691	1.01 (1.00-1.02)	**0.013**
Tumour Grade 1	REF		REF	
Tumour Grade 2	1.08 (0.49-2.38)	0.851	6.43 (1.52-27.26)	**0.012**
Tumour Grade 3	1.00 (0.43-2.34)	0.996	9.70 (2.27-41.41)	**0.002**
Lymphovascular invasion	1.61 (1.06-2.45)	0.025	1.22 (0.87-1.70)	0.250

All variables shown above were entered into the multivariate
NB: site of treatment has been hidden as it did not affect time to recurrence and was not significant

Supplementary Table 3: Multivariable and univariable logistic regression for margin involvement (close/involved vs. clear) in GM BCS patients only. Odds ratio (OR) greater than one implies higher odds of having close/involved margins. (1982 entered into the analyses excluding missing cases)

**Variable**	**Multivariate OR (95% CI)**	**p**
Age at Diagnosis	1.01 (1.00-1.02)	0.098
Radiotherapy	1.11 (0.77-1.60)	0.563
Chemotherapy	0.83 (0.61-1.12)	0.217
Hormone therapy	1.23 (0.75-2.04)	0.414
Herceptin therapy	1.54 (0.70-3.40)	0.285
Symptomatic vs. screening	1.73 (1.32-2.28)	**<0.001**
ER Positive	1.03 (0.23-4.57)	0.968
PR Positive	1.01 (0.75-1.37)	0.941
HER2 Positive	0.52 (0.20-1.38)	0.192
Molecular Subtype		
ER Positive HER2 Negative	REF	
ER Positive HER2 Positive	1.37 (0.73-2.56)	0.329
HER2 Enriched ER Negative	1.67 (0.29-9.62)	0.566
Triple Negative	1.32 (0.28-6.21)	0.728
T-Stage 1 *(0-2cm)*	REF	
T-Stage 2 *(2.1cm-5cm)*	1.25 (0.90-1.75)	0.189
T-Stage 3 *(>5cm)*	0.65 (0.19-2.28)	0.502
Number of positive nodes		
Negative (0)	REF	
1-3	0.98 (0.74-1.30)	0.873
4-9	0.80 (0.43-1.48)	0.472
10+	0.87 (0.38-1.98)	0.737
Size (mm)	1.01 (0.99-1.03)	0.381
Tumour Grade 1	REF	
Tumour Grade 2	1.46 (1.13-1.89)	**0.003**
Tumour Grade 3	1.44 (1.03-2.02)	**0.034**
Lymphovascular Invasion	0.97 (0.74-1.28)	0.832

NB: site of treatment has been hidden as it was not significant
All variables shown above were entered into the multivariate

Supplementary Table 4: Absolute recurrence rates and deaths in GM patients.

	All (370)			Breast Conservation (2030)			Mastectomy (1240)		
	Clear (2293)	*Close (302)*	*Involved (675)*	*Clear (1330)*	*Close (226)*	*Involved (474)*	*Clear (963)*	*Close (76)*	*Involved (201)*
**Local recurrence**	97 (4.2%)	14 (4.6%)	49 (7.3%)	40 (3.0%)	7 (3.1%)	32 (6.8%)	57 (5.9%)	7 (9.2%)	17 (8.5%)
**Distant Recurrence**	145 (6.3%)	23 (7.6%)	63 (9.3%)	38 (2.9%)	12 (5.3%)	28 (5.9%)	107 (11.1%)	14.5% (14.5%)	35 (17.4%)
**All deaths**	284 (12.4%)	44 (14.6%)	96 (14.2%)	96 (7.2%)	27 (11.9%)	58 (12.2%)	188 (19.5%)	17 (22.4%)	38 (18.9%)
**Deaths after recurrence**	128 (5.6%)	18 (6.0%)	47 (7.0%)	29 (2.2%)	11 (4.9%)	22 (4.6%)	99 (10.3%)	7 (9.2%)	25 (12.4%)

Supplementary Table 5: Absolute recurrence rates and deaths by T stage and nodal stage in BCS patients.

T- Stage	Local recurrence	Distant recurrence	Local and distant	p	Deaths	p
1 (n=1473)	33 (2.2%)	22 (1.5%)	7 (0.5%)	<0.001	103 (7.0%)	<0.001
2 (n=541)	29 (5.4%)	41 (7.6%)	6 (1.1%)		76 (14.0%)	
3 (n=19)	3 (15.8%)	2 (10.5%)	1 (5.3%)		3 (15.8%)	
Number of positive nodes						
0 (n=1664)	51 (3.1%)	32 (1.9%)	6 (0.4%)	<0.001	134 (8.1%)	0.002
1-3 (n=288)	10 (3.5%)	17 (5.9%)	7 (2.4%)		32 (11.1%)	
4-9 (n=53)	3 (5.7%)	12 (22.6%)	0		11 (20.8%)	
10+ (n=28)	1 (3.6%)	4 (14.3%)	1 (3.6%)		5 (17.9%)	

Radiotherapy was given to 93.5%, 89.1% and 737.7% of patients with T stage 1, 2 and 3 respectively, and 92.7%, 89.9%, 88.7% and 89.3% of patients with number of positive nodes 0, 1-3, 4-9 and 10+ respectively.

Supplementary table 6a: Margin location of closest margin and rates of involvement in BCS patients in GM patients

Which margin	BCS Involved n= 346 (17%)	LR in involved	DR in Involved	TOI n= 126(6.2%)	LR in TOI	DR in TOI
**Anterior**	127	4 (3.1%)	6 (4.7%)	50	3 (6.0%)	2 (4.0%)
**Inferior**	22	2 (9.1%)	0	15	3 (20%)	3 (20.0%)
**Lateral**	13	1 (7.7%)	2 (15.4%)	10	2 (20%)	1 (10.0%)
**Medial**	22	2 (9.1%)	4 (18.2%)	11	0	0
**Posterior**	137	12 (8.8%)	5 (3.6%)	31	2 (6.5%)	4 (12.9%)
**Superior**	25	1 (3.8%)	0	9	0	1 (11.1%)

In 472 patients with any BCS margin involved in the GM cohort, the margins involved are shown for involved margins <1mm and ToI. Excluding posterior margins, 64.4% of patients could have undergone re-excision for peripheral or anterior margin involvement after BCS .

In BCS patients radial margins were associated with LR and DR

Supplementary table 6b: Margin location of closest margin and rates of involvement in MX patients in GM patients

Which margin	Mx Involved n=137(11%)	LR in involved	DR in involved	TOI n=66(5.3%)	LR in TOI	DR in TOI
**Anterior**	28	3 (10.7%)	3 (10.7%)	12	1 (8.3%)	3 (25%)
**Inferior**	9	1 (11.1%)	0	2	0	1
**Lateral**	5	0	0	3	0	0
**Medial**	2	0	0	1	0	0
**Posterior**	87	7 (8.0%)	18 (20.7%)	45	3 (6.7%)	10 (22.2%)
**Superior**	6	0	0	3	2 (66.7%)	0

Mastectomy patients had final involved margins in 203(16.3%) cases of which 5.3% were Tumour on Ink (ToI)

In the Mastectomy patients LR and DR events were due to Anterior or posterior margin involvement except in 2 cases. The pattern of margin involvement and recurrence was different to that seen in BCS cases.

Supp Table 7: Cause-specific Cox proportional hazards model for time-to-local-recurrence (left) and time-to-distant-recurrence (right) in GM cases(BCS and Mx) with **Tumour on Ink (TOI) excluded (2993 patients in total in the analysis).**

Variable	Multivariate LR HR	p	Multivariate DR HR	p
Margin Clear>2mm	REF		REF	
Close 1.1-2mm	1.14 (0.64-2.02)	0.664	1.15 (0.72-1.85)	0.555
Involved Margin ≤1mm	1.56 (1.02-2.38)	**0.038**	1.22 (0.84-1.78)	0.289
Age at Diagnosis	0.99 (0.97-1.00)	0.058	1.01 (0.99-1.02)	0.317
Radiotherapy	0.97 (0.65-1.46)	0.885	0.81 (0.58-1.13)	0.216
Chemotherapy	0.90 (0.49-1.31)	0.381	0.99 (0.66-1.48)	0.955
Hormone therapy	0.62 (0.29-1.35)	0.230	0.44 (0.23-0.85)	**0.014**
Herceptin therapy	0.51 (0.14-1.90)	0.316	0.47 (0.18-1.21)	**0.117**
Symptomatic vs. screening	2.15 (1.32-3.49)	**0.002**	1.63 (1.10-2.43)	0.016
ER Positive	0.38 (0.05-2.90)	0.352	1.10 (0.15-8.33)	0.927
PR Positive	0.63 (0.37-1.05)	0.078	0.70 (0.47-1.05)	0.082
HER2 Positive	3.76 (0.57-24.87)	0.169	1.49 (0.35-6.36)	0.589
Molecular Subtype				
ER Positive HER2 Negative	REF		REF	
ER Positive HER2 Positive	0.51 (0.12-2.25)	0.377	1.37 (0.42-4.45)	0.606
Her2 Enriched ER Negative	0.08 (0.01-1.09)	0.058	0.38 (0.03-4.29)	0.435
Triple Negative	0.46 (0.06-3.74)	0.470	0.73 (0.10-5.61)	0.763
T-Stage 1 *(0-2cm)*	REF		REF	
T-Stage 2 *(2.1cm-5cm)*	1.47 (0.94-2.30)	0.091	1.49 (1.03-2.16)	**0.035**
T-Stage 3 *(>5cm)*	1.28 (0.50-3.28)	0.611	1.45 (0.76-2.77)	0.262
Number of positive nodes				
Negative (0)	REF		REF	
1-3	1.59 (1.05-2.42)	**0.028**	1.77 (1.22-2.56)	**0.002**
4-9	1.42 (0.71-2.86)	0.325	3.60 (2.27-5.73)	**<0.001**
10+	1.65 (0.74-3.67)	0.223	4.18 (2.52-6.933)	**<0.001**
Size (mm)	1.01 (0.99-1.02)	0.316	1.02 (1.01-1.02)	**0.001**
Tumour Grade 1	REF		REF	
Tumour Grade 2	1.10 (0.57-2.13)	0.782	3.60 (1.42-9.12)	**0.007**
Tumour Grade 3	1.17 (0.56-2.42)	0.676	5.16 (1.99-13.35)	**0.001**
Lymphovascular invasion	1.78 (1.20-2.63)	**0.004**	1.27 (0.92-1.74)	0.142

All variables shown above were entered into the multivariate
NB: site of treatment has been hidden as it did not affect time to recurrence and was not significant

Supp Table 8:Cause-specific Cox proportional hazards model for time-to-local-recurrence (left) and time-to-distant-recurrence (right) in GM BCS cases with **Tumour on Ink (TOI) excluded for BCS (1857 patients in total in the analysis).**

**Variable**	**Multivariate LR HR**	**P**	**Multivariate DR HR**	**P**
Margin Clear>2mm	REF	0.063	REF	0.287
Close 1.1-2mm	0.90 (0.39-2.07)	0.802	1.57 (0.78-3.16)	0.211
Involved Margin ≤1mm	1.90 (1.07-3.31)	**0.027**	1.46 (0.80-2.66)	0.214
Age at Diagnosis	0.99 (0.96-1.01)	0.228	0.99 (0.97-1.01)	0.280
Radiotherapy	1.87 (0.87-3.99)	0.107	1.92 (0.93-3.97)	0.077
Chemotherapy	1.27 (0.60-2.76)	0.537	1.51 (0.74-3.09)	0.254
Hormone therapy	2.89 (0.99-8.47)	0.053	5.01 (2.06-12.15)	**<0.001**
Herceptin therapy	0.67 (0.05-9.77)	0.766	1.63 (0.24-11.14)	0.618
Symptomatic vs. screening	1.46 (0.77-2.78)	0.244	1.17 (0.63-2.20)	0.617
ER Positive	0.26 (0.08-0.85)	**0.026**	0.26 (0.09-0.72)	**0.009**
PR Positive	3.08 (1.64-5.79)	**<0.001**	1.92 (0.99-3.69)	0.051
HER2 Positive	2.32 (0.16-33.50)	0.536	0.81 (0.13-5.11)	0.824
T-Stage 1 *(0-2cm)*	REF	0.078	REF	0.237
T-Stage 2 *(2.1cm-5cm)*	2.31 (1.03-5.20)	**0.042**	1.56 (0.70-3.47)	0.272
T-Stage 3 *(>5cm)*	1.50 (0.10-21.76)	0.767	0.52 (0.04-7.21)	0.626
Number of positive nodes				
Negative (0)	REF	0.395	REF	**<0.001**
1-3	1.45 (0.77-2.74)	0.255	2.44 (1.32-4.52)	**0.005**
4-9	1.25 (0.36-4.36)	0.724	5.57 (2.49-12.48)	**<0.001**
10+	0.29 (0.03-2.47)	0.258	1.71 (0.45-6.42)	0.432
Size (mm)	0.99 (0.95-1.04)	0.786	1.02 (0.98-1.07)	0.289
Tumour Grade 1	REF	0.965	REF	**0.022**
Tumour Grade 2	1.09 (0.48-2.51)	0.835	3.48 (0.80-15.17)	0.098
Tumour Grade 3	1.14 (0.43-3.04)	0.791	6.64 (1.45-30.43)	**0.015**
Lymphovascular invasion	1.63 (0.88-3.02)	0.124	0.97 (0.53-1.77)	0.916

All variables shown above were entered into the multivariate

NB: site of treatment has been hidden as it did not affect time to recurrence and was not significant

Supplementary Table 9: UK National Cancer registry: Multivariable analysis of Factors affecting Breast Cancer Death in women aged 20-112 years.

**Factor**	**Frequency**	**n**	**Margins<1mm**	**Multivariate**	**95% CI**		**P Value**
Margins	Margins >1mm	27644	0.0%	REF			
	"Clear"	3940	0.0%	0.90	0.80	1.02	0.091
	Margin<1mm	9210	100.0%	1.15	1.06	1.25	**0.001**
Age	increase/year	40794	22.6%	1.03	1.02	1.03	**<0.001**
Deprivation Status	Least (1)	9646	24.2%	1.01	0.90	1.12	0.902
	2	10066	23.3%	1.03	0.93	1.14	0.605
	3	8988	22.3%	REF			
	4	6851	20.9%	1.21	1.08	1.35	**0.001**
	Most dep (5)	5243	21.1%	1.30	1.15	1.46	**<0.001**
Stage of Ca	Stage 1	21287	21.1%	REF			
	Stage 2	16079	24.1%	2.07	1.88	2.28	**<0.001**
	Stage 3	3428	24.9%	5.33	4.78	5.95	**<0.001**
Diagnostic route	Screen Detected	16107	20.5%	REF			
	Symptomatic	15053	23.4%	1.81	1.62	2.01	**<0.001**
	Unknown	9634	24.8%	2.25	2.02	2.52	**<0.001**
Primary treatment	Breast Conserving Surgery (BCS)	23335	24.9%	REF			
	Neoadjuvant chemo to BCS	370	27.3%	2.46	1.93	3.14	**<0.001**
	Neoadjuvant ET followed by BCS	588	27.7%	1.26	0.94	1.68	0.119
	Mastectomy	10213	16.9%	1.36	1.25	1.48	**<0.001**
	Neoadjuvant chemotherapy followed by MX	504	16.1%	2.94	2.45	3.53	**<0.001**
	Neoadjuvant ET followed by MX	395	16.2%	1.77	1.38	2.25	**<0.001**
	Unknown/other	5389	23.4%	1.05	0.92	1.18	0.480
Grade	Grade 1	6822	18.9%	REF			
	Grade 2	20150	22.3%	2.45	1.97	3.05	**<0.001**
	Grade 3	13325	24.9%	6.40	5.16	7.93	**<0.001**
	Unknown grade	497	21.1%	3.92	2.66	5.78	**<0.001**

All variables shown above were entered into the multivariate. Data for primary treatment was missing from some older patients but all patients not receiving surgery first(BCS or Mastectomy) were excluded from the analysis.

Supplementary Table 10: National Cancer registry: Multivariable analysis of Factors affecting Breast Cancer Death in women aged 25-70 years who presented symptomatically and received BCS as a part of their treatment.

Factor	Frequency	N	Margins<1mm	Multivariate	95% CI		P Value
Margins	Margins >1mm	3716	0.0%	REF			
	"Clear"	511	0.0%	0.84	0.57	1.24	p=0.387
	Margin<1mm	1673	100.0%	1.33	1.08	1.63	**p=0.007**
Age	Age increase/year	5900	28.4%	1.03	1.01	1.04	**p<0.001**
Deprivation Status	Least (1)	1435	30.9%	1.14	0.85	1.52	p=0.384
	2	1465	29.8%	1.04	0.77	1.39	p=0.810
	3	1307	27.8%	REF			
	4	911	24.4%	1.36	0.99	1.85	p=0.054
	Most deprived (5)	782	26.6%	1.25	0.90	1.75	p=0.187
Stage of Ca	Stage 1	3013	25.4%	REF			
	Stage 2	2578	30.6%	2.01	1.59	2.53	**p<0.001**
	Stage 3	309	38.2%	5.08	3.73	6.90	**p<0.001**
Grade	1	829	24.5%	REF			
	2	2610	27.4%	3.67	1.70	7.91	**p<0.001**
	3	2405	30.9%	11.28	5.31	23.98	**p<0.001**
	Unknown	56	21.4%	5.53	1.43	21.41	**p=0.013**

All variables shown above were entered into the multivariate. Data from primary treatment was missing from some older patients but all patients not receiving surgery first(BCS or Mastectomy) were excluded from the analysis.

Supplementary File 11 Independent Patient Cancer Voice advocacy group survey

Jacqui Gath (ICPV PPI lead).sent questions to a UK IPCV patient group .

A simple ad-hoc questionnaire was sent to members of Independent Cancer Patient Voice(ICPV) who had experienced breast cancer by the PPI member of the team. There was no selection of members.The questionnaire requested the patient views about the importance of preventing metastasis versus achieving more pleasing effects of surgery. All 15 women who replied voluntarily asserted that it was more important to them to avoid distant recurrence than insist on a more pleasing cosmetic result. Two other respondees had experienced other cancers and were adamant that a larger margin was acceptable to avoid further disease.

Conclusions and summary

The clear message received from the Independent Cancer Patient Voice advocacy group was that decisions regarding margin widths and re-excision must be consensual between patient and surgeon. Unanimously they preferred oncological safety and margin clearance above cosmetic outcomes. The importance of patient voice cannot be overestimated when it comes to “choice” of outcomes such as this
